# Find Me If You Can: First Clinical Experience Using the Novel CARTOFINDER Algorithm in a Routine Workflow for Atrial Fibrillation Ablation

**DOI:** 10.3390/jcm10132979

**Published:** 2021-07-02

**Authors:** Robin Unland, Leonard Bergau, Mustapha El Hamriti, Denise Guckel, Misagh Piran, Thomas Fink, Vanessa Sciacca, Hermann Köerperich, Mikhail Chmelevsky, Guram Imnadze, Moneeb Khalaph, Martin Braun, Philipp Sommer, Christian Sohns

**Affiliations:** 1 Clinic for Electrophysiology, Herz- und Diabeteszentrum NRW, Ruhr-Universität Bochum, Georgstr. 11, 32545 Bad Oeynhausen, Germany; robin.unland97@gmail.com (R.U.); lbergau@hdz-nrw.de (L.B.); melhamriti@hdz-nrw.de (M.E.H.); dguckel@hdz-nrw.de (D.G.); tfink@hdz-nrw.de (T.F.); vsciacca@hdz-nrw.de (V.S.); gimnadze@hdz-nrw.de (G.I.); mkhalaph@hdz-nrw.de (M.K.); mbraun@hdz-nrw.de (M.B.); psommer@hdz-nrw.de (P.S.); 2 Nuclear Medicine and Molecular Imaging, Herz- und Diabeteszentrum NRW, Institute for Radiology, Ruhr-Universität Bochum, 32545 Bad Oeynhausen, Germany; mpiran@hdz-nrw.de (M.P.); hkoerperich@hdz-nrw.de (H.K.); 3 Almazov National Medical Research Center, Department of Electrocardiology, 197341 Saint-Petersburg, Russia; boxmch@gmail.com; 4 World-Class Scientific Center, Saint-Petersburg Electrotechnical University, 196128 Saint-Petersburg, Russia

**Keywords:** atrial fibrillation, catheter ablation, panoramic mapping, CARTOFINDER

## Abstract

Aims: The CARTOFINDER module allows for simultaneous and automated detection of repetitive focal and rotational activations in patients with atrial arrhythmias. This study aimed to validate the CARTOFINDER algorithm for the detection of potential drivers for atrial fibrillation (AF) and to access their potential impact on individual arrhythmia substrates. Methods: Fifty consecutive patients underwent AF ablation for persistent AF (PERS), using a 3D-mapping system with the integrated CARTOFINDER module. Regions of interest (ROIs) were identified before and after ablation, and their spatial and temporal relationship was correlated with areas of fibrosis. Results: Procedural success was achieved in all patients and 42% received ablation beyond pulmonary vein isolation (PVI). AF termination was observed in 6 patients (12%). The mean procedure duration was 134 ± 29 min. ROIs were revealed in all patients (mean *n* = 77 ± 52) and there was no statistical evidence for a predilection site. There was no significant anatomical correlation between ROIs and bipolar low voltage. Remapping confirmed the elimination of ROIs in relation to the individual ablation site, a limited reproducibility of rotational ROIs and persistent focal activity over time in some anatomical segments. ROIs were not a predictor for AF recurrence during following ablation. Conclusions: CARTOFINDER mapping can be integrated into a routine workflow for AF ablation. ROIs could be discriminated in all patients and an ablation effect was observed in some patients, whereas persistent activity was found in certain anatomical segments, even after ablation. ROIs might be an additional ablation target when we are able to understand the individual substrate.

## 1. Introduction

Catheter ablation for persistent atrial fibrillation (AF) is challenging and associated with limited outcomes [1]. The mechanisms initiating and perpetuating AF are still not completely understood and therefore, ablation strategies are heterogeneous. Several observations have led to individual mechanistic insights in AF management and arrhythmia-associated cardiac remodeling which emphasize the need for personalized paths in AF therapy [2,3]. Despite improvements in ablation technology, imaging modalities, and ablation techniques, the recurrence of atrial arrhythmias after AF ablation still remains frequent [1,4]. Advances in mapping technologies have allowed for more detailed approaches, including panoramic and body-surface mapping for potential individual focal AF driver or rotational activity [5,6,7,8]. The elimination of these phenomena has also been linked to higher ablation success [9,10]. Recently, the CARTOFINDER module was introduced as a potential novel approach to improve the understanding of individual arrhythmia substrates based on panoramic mapping. On the basis of this technology, focal and rotation activity can be revealed during sustained atrial arrhythmias and directly visualized inside the electroanatomical mapping system to guide ablation.

This study sought to use the CARTOFINDER algorithm to detect and characterize focal and rotational activity in persistent AF (PERS) and to examine the individual relationship between these findings and fibrotic atrial tissue.

## 2. Methods

A total of 50 consecutive patients with drug-refractory PERS were included in this prospective observational analysis. AF was defined as persistent if AF was continuously sustained beyond 7 days, including episodes terminated by cardioversion after ≥7 days [1]. All patients were ablated at our institution. Written informed consent was obtained from each patient, and the current study complies with the Declaration of Helsinki and was approved by the institutional review board.

**Figure 1:**
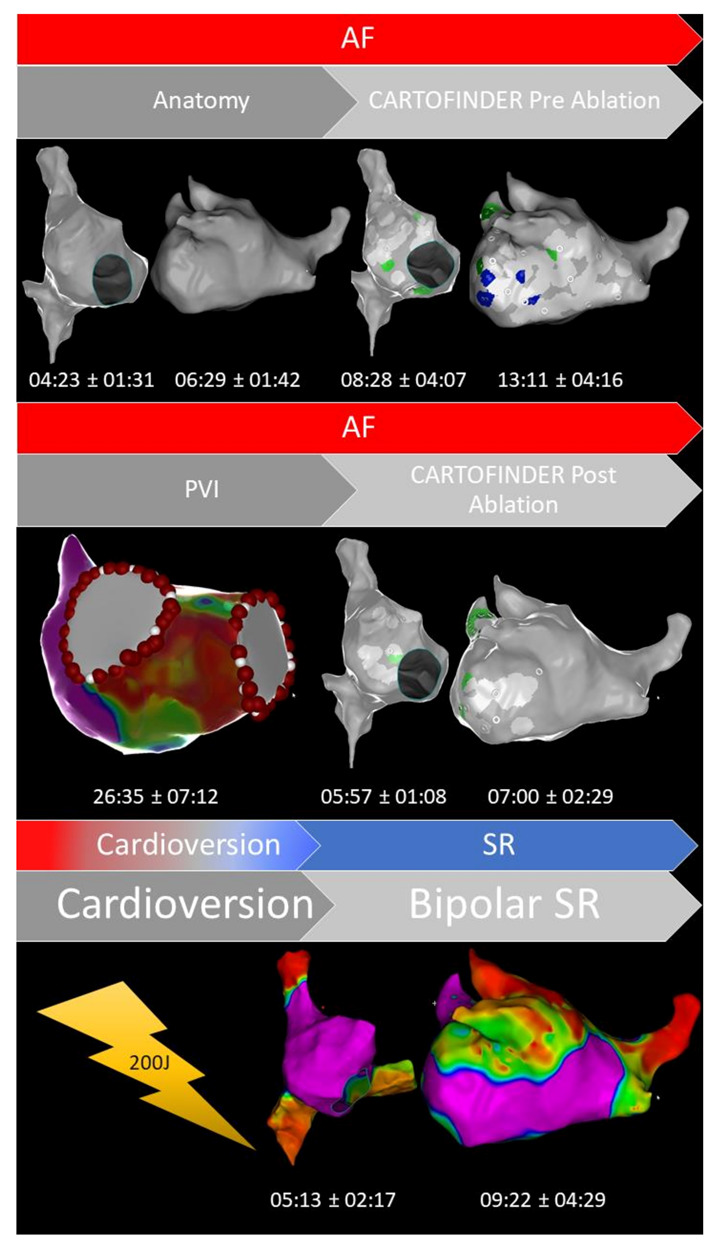
Representative example of our CARTOFINDER workflow (focal activity, green color; rotational activity, blue color) in patients with persistent atrial fibrillation (AF, atrial fibrillation; PVI, pulmonary vein isolation; SR, sinus rhythm). The mean time for each workflow-step is visualized in minutes ± standard deviation.

The complete workflow is summarized in Figure 1. LA thrombus formation was ruled out prior to ablation in all patients. All procedures were performed on uninterrupted oral vitamin K anticoagulants with a target INR of 2.0–3.0 on the day of the procedure; direct oral anticoagulants (DOACs) were discontinued the day of the procedure and resumed the same day after ruling out pericardial effusion. Catheter ablation was performed under deep sedation with bolus of midazolam and fentanyl and a continuous infusion of propofol. A 6F diagnostic catheter was inserted distally into the coronary sinus (CS) via the right femoral vein. Double transseptal puncture was performed (TSP), using 8.5F SL1 sheaths (SJM, St. Paul, MN, USA) and a modified Brockenbrough technique. Unfractionated heparin was administered according to the patient’s weight to maintain an activated clotting time (ACT) of ≥300 s.

### 2.1. CARTOFINDER Analysis

**Figure 2:**
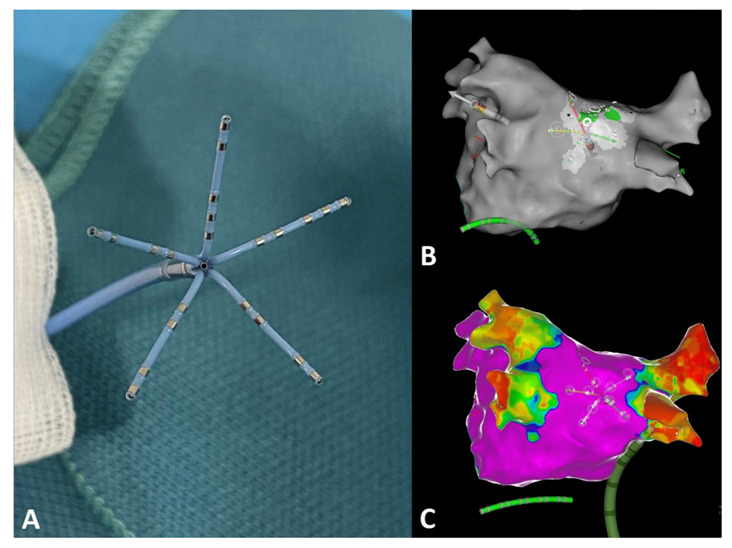
CARTOFINDER-guided mapping for regions of interest (ROI) was conducted automatically, utilizing the multipolar PentaRay catheter. (**A**) Photographic representation of the PentaRay catheter with its five splines. (**B**) Representative example for CARTOFINDER-guided mapping for ROIs at the posterior left atrial wall, using the PentaRay mapping catheter. (**C**) Bipolar high-density mapping of the left atrium (**B**,**C**) posterior–anterior view.

**Figure 3:**
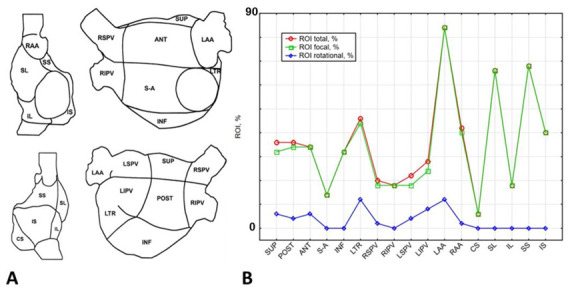
(**A**) Schematic reconstruction from of the right (RA) and left atrium (LA) in anterior/posterior (AP) and posterior/anterior (PA) view. The RA was divided into the following segments: right atrial appendage (RA RAA), coronary sinus ostial area (RA CS), superior lateral (RA SUP LAT), inferior lateral (RA INF LAT), superior septal (RA SUP SEP), inferior septal (RA INF SEP), superior cava vein (SCV), and inferior cava vein (ICV). The LA was divided into the following segments: superior (LA SUP), posterior (LA POST), anterior (LA ANT), inferior (LA INF), lateral (LA LAT), right superior pulmonary vein (LA RSPV), right inferior PV (LA RIPV), left superior PV (LA LSPV), left inferior PV (LA IPV), septal-anterior (LA SEPT ANT), and left atrial appendage (LAA). (**B**) Distribution of Regions of Interest (ROI; red color, focal + rotational; green color, focal; blue color, rotational) from CARTOFINDER mapping separated per atrial segment.

**Figure 4:**
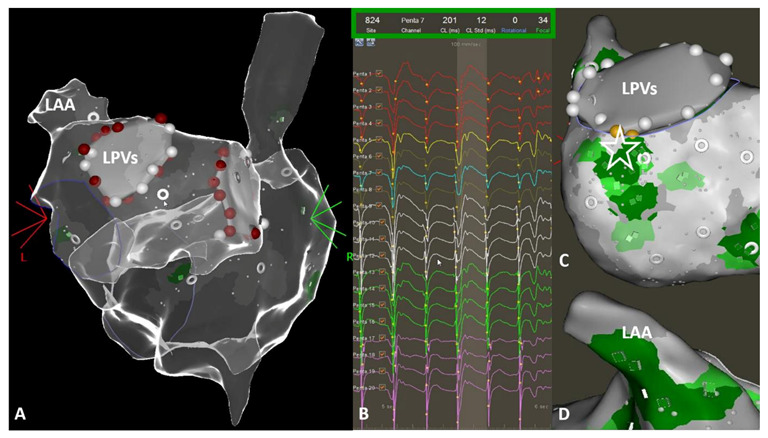
(**A**,**C**) Typical example for focal activities (green color) revealed from CARTOFINDER mapping of the right and left atrium and left atrial appendage. (**D**) The grey ring represents the center of a mapping position, using the PentaRay catheter. The light grey surface around the ring visualizes the coverage of the LA wall surface with the five PentaRay splines. (**B**) Focal activation is detected by identifying an S wave in the unipolar electrograms. If S wave patterns preceding activity on neighboring electrodes are detected during at least two consecutive atrial cycles, the site is designated as a focal source.

**Figure 5:**
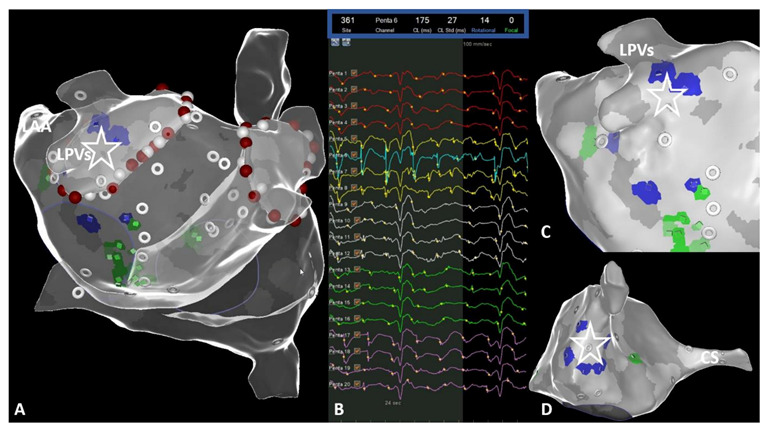
(**A**,**C**,**D**) Typical example for rotational activities (blue color) revealed from CARTOFINDER mapping of the left atrium. (**B**) Rotational activation is detected by identifying pan-systolic activation occurring in consecutive electrodes. A pan-systolic activation wave is defined as a series of electrograms in consecutive electrodes, occupying more than 50% of the local cycle length with a distance of less than 20 mm between the starting and ending points of the wave. Two or more such pan-systolic activations occurring are defined as a rotational activation.

**Figure 6:**
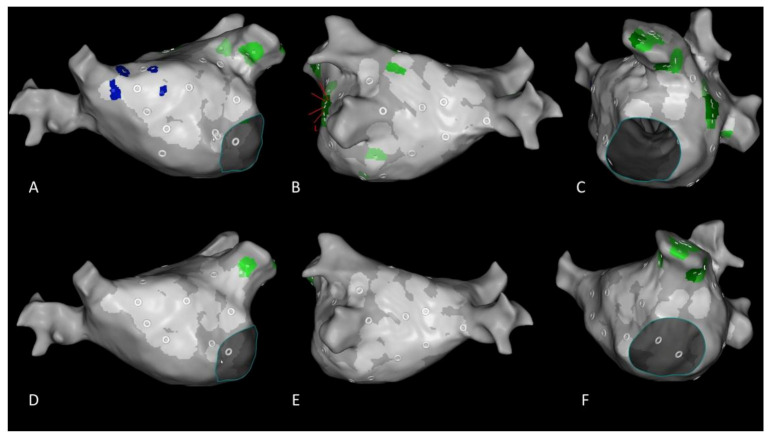
Representative image to demonstrate and the temporal stability of region of interests (ROIs) from CARTOFINDER mapping. Upper panels: Findings from CARTOFINDER mapping prior to ablation. Lower panels: Findings from CARTOFINDER mapping after ablation. (**A**,**C**,**D**,**F**) Persistent focal activity (green spots) in the left atrial appendage, independent of pulmonary vein isolation and left atrial substrate modification. (**A**,**D**) In contrast, the rotational activity (blue spots) disappeared after catheter ablation. (**B**,**E**) This was also the case for focal activity (green spots) around the left sided pulmonary veins.

The CARTOFINDER algorithm has been reported before [11]. In contrast to previous examinations, mapping for regions of interest (ROI) was conducted automatically, utilizing the multipolar PentaRay catheter (Figure 2; PentaRay; Biosense-Webster Inc., Diamond Bar, CA, USA) and in real time as part of the routine clinical workflow, without the need for repeat off-line modification. Briefly, during the mapping for individual ROIs, the system records 30 s of unipolar signals obtained from a multipolar mapping catheter by referencing to Wilson’s Central Terminal and filtering between 0.05 and 500 Hz. The signals then undergo processing whereby ventricular far field signals are first filtered. Afterward, for each unipolar signal, 2 bipolar signals are created by pairing the electrode with the nearest 2 electrodes from the multipolar mapping catheter. A bipolar electrogram window is then applied to the unipolar signals that range from earliest onset to the latest offset of the 2 bipolar electrograms. Atrial signals within the bipolar electrogram window are then annotated automatically by the software, whereas areas outside this window are excluded. Atrial signals are annotated at the point of the maximum negative slope, using wavelet analysis. The local activation time (LAT) is then determined from the annotated unipolar signals. CARTOFINDER creates activation maps during a 250 ms window, referencing each electrogram relative to all the others in the RA or LA. This time window then moves through the 30 s recording to show a changing activation map over time [11]. Multiple biatrial 30 s recordings for ROIs were taken, using the CARTOFINDER algorithm in AF to cover the complete surface of the RA and LA. The RA and LA was divided into 17 segments to compare the presence of ROIs with the amount and distribution of bipolar low voltage based on these segments as demonstrated in Figure 3, and an automated algorithm was used to reveal focal (Figure 4) and rotational (Figure 5) activations at each site during the 30 s recording period in AF. Repeat CARTOFINDER mapping was performed after PVI as well as additional substrate modification ablation to exanimate potential ablation effects on ROIs and their stability over time (Figure 6).

### 2.2. Ablation Procedure

Catheter ablation was performed with an open-irrigated tip catheter (Thermocool SmartTouch SF, Biosense Webster Inc.). After reconstruction of the LA, each pulmonary vein (PV) ostium was identified by selective PV angiography, and ablation was performed. Irrigated RF was delivered, targeting a maximum temperature of 43 °C, a maximum power level of 35 W and an infusion rate of 20 mL/min. PVI was guided by ablation index (AI) values (anterior, roof, bottom: 550; posterior: 400), targeting an interlesion distance of ≤6 mm. Following PVI, additional LA substrate modification was performed in the case of individual arrhythmia substrates revealed from the bipolar voltage mapping. In patients with the coincidence of typical atrial flutter, cavo-tricuspid isthmus ablation was performed at the end of the procedure. In the case of linear lesion sets, a conduction block along the lines was validated in sinus rhythm. Procedural success was subsequently reconfirmed after a minimum waiting period of 30 min.

### 2.3. Ultra High Density Mapping

After restoration of the sinus rhythm, the multipolar mapping catheter (PentaRay, Biosense-Webster Inc., Diamond Bar, CA, USA) was used to gain information about the localization and distribution of the bipolar low voltage in the RA and LA wall. The same anatomical segments were used as described above (Figure 3). Ultra high density mapping, aiming for >1000 mapping points per atrium, was conducted in all patients. For the atrial voltage maps, the bipolar voltage reference interval was set between 0.05 and 0.5 mV. The amount of LA bipolar low voltage (total and per segment) was measured, using the area measurement tool.

### 2.4. Follow-Up

All patients were followed in our outpatients’ clinic 3 and 6 months after ablation. At each visit, they were asked for any symptoms suggestive of arrhythmia recurrence or discomfort during respiration. Moreover, a 72 h Holter ECG was routinely performed at each presentation to monitor arrhythmia recurrence and burden, as well. Following a 3 month blanking period, recurrence was defined as any symptomatic episode of AT/AF lasting >30 s.

### 2.5. Statistical Analysis

The preliminary exploratory analysis was conducted to evaluate the distribution of different data; an initially *p* value < 0.05 was considered to be statistically significant. Patient clinical characteristics are reported with descriptive statistics as mean and standard deviation, while categorical data are presented as numbers and percentages. Detailed analysis of the correlation between ROIs and bipolar low-voltage was conducted using the Spearman’s rank correlation test. The Mann–Whitney U test was performed to compare independent samples, including patients’ characteristics with the number of ROIs and their distribution. Linear relationships between individual predictors and certain continuous dependent quantitative variables were evaluated, using linear regression analysis followed by detailed evaluation of 2D scatter plots.

The amount of bipolar low voltage was compared to AF recurrence, using ROC analysis with detailed evaluation of 95% confidence intervals. Furthermore, a general discriminant analysis followed by ROC analysis was used as a multivariate technique to evaluate the different amounts of bipolar low-voltage and number of ROIs as predictors for AF recurrence. The evaluation and interpretation of the obtained classification functions were carried out, taking into account the level of their statistical significance, followed by the canonical analysis and plotting of these functions. The classification matrices were also analyzed in detail to assess the quality of each obtained function. All comparisons were made in accordance with the cross-sectional study design and descriptive study guidelines. Finally, a *p*-value < 0.01 was considered to be statistically significant, due to the Bonferroni correction. Comprehensive statistical analysis was performed using Statistica v.10 (Statsoft Inc., USA) and SPSS v.23 (IBM Corp., USA).

## 3. Results

### 3.1. Patient Characteristics

A total of 50 patients (median age 67 years; range 34–85 years) with PERS underwent de novo AF ablation in conjunction with CARTOFINDER mapping for ROIs. The median AF duration was 30 ± 25 months. The clinical baseline characteristics of the study population are depicted in Table 1.

### 3.2. Procedural Data

Acute PVI was achieved in all patients. In 42% of these patients, additional substrate modification in the LA was performed. Substrate modification was based on the individual distribution of bipolar low voltage areas and included posterior wall isolation (box-lesion, roof and bottom line) in 4 patients (8%), a left atrial anterior line from the mitral valve annulus to the right superior PVs in 12 patients (24%), a single roof line in 3 patients (6%), a linear lesion set across the lateral mitral valve isthmus in 1 patient (2%) and a LAA isolation in one patient (2%). AF termination was observed in 6 patients (12%). In 3 patients, AF termination was achieved during isolation of the left-sided PVs; in another 3 patients, AF changed to left atrial macro-reentrant tachycardia (LAMRT) during ablation. AF termination was observed anterior–inferior to the left inferior PV in one patient; in the remaining two patients, AF termination resulted in isolation of the right-sided PVs. In all cases, LAMRT was mapped and successfully ablated inside the LA by application of an anterior line. All patients with AF termination during ablation had focal ROIs (with a CL ranging from 161 ms to 296 ms) in close relationship to the wide circumferential ablation line. Programmed atrial stimulation (including high-rate stimulation) failed to re-induce any atrial tachyarrhythmia after ablation. Of note, an ablation effect on the presence and stability of ROIs after ablation could not be analyzed in these patients.

The mean procedure duration (skin-to-skin) was 134 ± 29 min. The entire workflow and the procedural data are summarized in Table 2 and Figure 1. A mean number of 77 ± 52 ROIs was revealed from CARTOFINDER (RA 32 ± 19; LA 45 ± 33). A mean number of 22 stable mapping positions for ROIs was required (RA: 9; LA: 13) to cover the whole biatrial surface with the multipolar mapping catheter. There was no statistically significant evidence for a predilection site in terms of focal or rotational activity (*p* = 0.5). In the LA, focal activity was predominantly observed inside the LAA (*n* = 42; 84%) and in close relationship to the left PV ostia (inferior and lateral; LIPV *n* = 12; 24%; LTR *n* = 22, 44%). Furthermore, we mapped an increased amount of focal activity among the superior, posterior and anterior segments of the LA (SUP *n* = 16; 32%; POST *n* = 17; 34%; ANT *n* = 17; 34%). The majority of ROIs in terms of rotational activity was also found in the left-lateral segments, including the LPVs, LA/LAA ridge and mitral valve annulus (LAA *n* = 6, 12%; LTR *n* = 6, 12%; LIPV *n* = 4, 8%). In the RA, the majority of ROIs (focal + rotational) was found at the superior aspects (superior–septal; SS *n* = 34; 68% and superior–lateral; SL *n* = 33; 66%), inside the inferior–septal segment; (IS *n* = 20; 40%) and in close relationship to the RA appendage (RAA *n* = 21; 42%).

The mean amount of bipolar low voltage was 21 ± 12% for the RA and 35 ± 13% for the LA. The segments with the highest amount of bipolar low voltage in the LA were located in close relationship to the PVs (RSPV: 87 ± 13%; RIPV 79 ± 21%; LSPV 82 ± 18%; LIPV 82 ± 18%), anterior (LA ANT: 26 ± 26%) and lateral (LA LTR: 26 ± 22%). For the RA, the highest amount of bipolar low voltage was detected inferior–lateral (RA IL: 31 ± 30%) and septal in close relationship to the coronary sinus (RA CS: 29 ± 28%), respectively. There was no statistically significant anatomical correlation between ROIs and bipolar low voltage in the RA and LA.

### 3.3. Ablation Effect and Stability of ROIs over Time

During re-mapping for ROIs after ablation (Figure 5; duration between 1st and 2nd CARTOFINDER mapping: 61 ± 8 min), we observed the elimination of all ROIs close to the ablation set for PVI. In addition, the rotational activity revealed at pre-ablation mapping was only re-identified at repeat mapping in one patient (location: LAA). Repeat mapping discriminated new ROIs for rotational activity following ablation in 7 patients (14%; anatomical location; LA: LTR, LAA; RA: SL, SS, IS). Of note, we found persistent focal activity in the LAA (*n* = 33; 66%) and RA, especially at the superior areas of the RA, the RA SL (*n* = 18; 36%) and RA SS (*n* = 19; 38%) after AF ablation.

### 3.4. Follow-Up Data and Complications

No procedure relating major complications (requiring intervention) were observed. The patient with LAA isolation (LAAi) received an uneventful LAA occlusion device six weeks after ablation. During a mean follow-up of 6.4 ± 3.6 months, 41/50 (82%) of patients remained free from any arrhythmia recurrence. The amount of bipolar low voltage was not a significant predictor for AF/AT recurrence in univariate analysis (*p* = 0.501). This was also true for ROIs (*p* > 0.05). Focusing on arrhythmia recurrence, all patients (*n* = 9; 18%) presented with AF. Two patients with arrhythmia recurrence underwent repeat ablation. PV reconnection was found in 1 patient (2%) and another patient (2%) had AF recurrence, despite persistent PVI. In these patients, substrate modification was performed based on bipolar low voltage mapping.

## 4. Discussion

### 4.1. Major Findings

This prospective observational study had three major findings: first, CARTOFINDER mapping can be easily integrated into a routine workflow for PERS ablation. Second, ROIs could be discriminated and visualized in all patients. Third, an ablation effect was observed on the presence of ROIs with a close relationship to the PVs, whereas persistent activity was found in the LAA and RA, even after ablation.

### 4.2. Benefits of CARTOFINDER Mapping in Persistent AF

Previous studies have reported high quality in terms of the sensitivity and specificity for ROIs based on CARTOFINDER mapping [11,12,13]. These studies were conducted with a previous software version of the algorithm requiring manual reannotation; mapping for ROIs was conducted utilizing a basket catheter. In addition, the data were not acquired as part of a routine clinical workflow for AF ablation in patients with PERS. Our data indicate that mapping and ablation could be performed with only a limited increase in procedure time (Table 2, Figure 1). Of note, multiple electroanatomical maps were generated and individual ROIs were revealed in all patients. Procedure duration reported from AF ablation procedures based on other mapping systems, including FIRM- or bodysurface-mapping, were significantly longer [10,14] and the procedure itself was particularly complicated due to technical issues between imaging, mapping catheter, analysis software and the integration into the electroanatomical mapping system. In this context, an important finding from this analysis is the observation that focal ROIs can be successfully targeted during wide area circumferential ablation for PVI or during substrate modification, sometimes resulting in AF termination or otherwise remaining stable over time (Figure 5). Consequently, these ROIs should potentially be eliminated in addition to PVI. A comparable effect of focal sources was reported previously [10,14,15]. In contrast to focal ROIs, rotational findings from CARTOFINDER mapping were not reproducible during remapping. One may speculate that these findings represent a temporal phenomenon requiring an endo- or epicardial arrhythmia substrate in terms of arrhythmogenesis [7,10,16]. Conflicting data exist, focusing on the individual temporal and spatial relationship between atrial fibrosis or scar tissue and ROIs in terms of focal and rotational activity. We performed bipolar voltage mapping to analyze a potential anatomical relationship between fibrotic atrial tissue and the presence of ROIs as part of our routine AF ablation workflow (Figure 1). This might be helpful to develop specific ablation strategies for ROIs from CARTOFINDER mapping. However, we found no significant relationship between the amount and distribution of bipolar low voltage and focal or rotational ROIs. Consequently, the presence and location of ROIs seem to be individual and not predictable from voltage mapping or any certain characteristics. As we need personalized paths in AF management, we might require a separated mapping approach for ROIs as part of a routine workflow for PERS, and ablation strategies for these ROIs need to be derived from their temporal and spatial distribution.

### 4.3. Regions of Interest—New Targets for AF Ablation

AF termination is a good predictor for freedom from arrhythmia recurrence after ablation; we observed this phenomenon in 6 patients (12%). In all of these patients, ROIs were detected in close relationship to the specific sites where ablation resulted in AF termination. Cochet et al. reported that the number of re-entrant regions during AF related to the extent of fibrosis from Magnetic Resonance Imaging (MRI). The authors demonstrated that the relationship between fibrosis and re-entrant regions affected the procedural outcomes and they reported AF termination following their Late Gadolinium Enhancement (LGE)-guided ablation approach in 65% of patients [10]. In this context, the spatial correlation between focal and rotational activity and fibrosis in AF is still debated. However, a growing body of evidence supports the role of fibrosis in anchoring rotational activity during AF [10,16,17,18]. Fibrosis imaging based on LGE-MRI was not performed in our study but will be the subject of future analysis. We performed bipolar voltage mapping, aiming for > 1000 mapping points per acquired atrial map. However, our results act in concert with recent studies showing that AF episodes are perpetuated by individual drivers in boarder zones between normal and scarred tissue [7,10,16,17]. These observations bring new insights into the mechanisms of PERS based on focal and rotations ROIs from CARTOFINDER at specific atrial locations, as they suggest that patient-specific characteristics of the atrial myocardium may provide an individual arrhythmia substrate. This may also explain why in some patients, the elimination of ROIs resulted in AF organization or termination. However, the limited number of patients with AF termination and the tailored ablation approaches beyond PVI do not yet allow a detailed analysis of the individual substrates and the relationship to findings from CARTOFINDER mapping. Therefore, additional research is needed to further access how focal and rotational activity anchors, and particularly to discriminate between, structural and functional arrhythmia mechanisms.

### 4.4. Can CARTOFINDER Mapping Identify Responder to LAA Isolation?

LAAi might have beneficial effects on clinical outcome in selected patients with PERS [19], but the side effects of this intervention have to be considered. Therefore, it is desirable to identify patients with a high probability to respond to LAAi. Identifying propagation patterns based on focal and rotational activity in the LAA during AF might help to determine such suitable candidates. The LAA was analyzed using CARTOFINDER mapping in all patients. Our study demonstrates that the LAA was found to be the most frequent anatomical localization for ROIs (focal *n* = 42; 84%; rotational *n* = 6; 12%), even prior to ablation. Takahashi et al. reported that CARTOFINDER mapping more often identified focal activation than rotational activation in the LAA in persistent AF, and that focal ROIs were observed most frequently in the LAA [20]. Of note, after PVI and substrate modification, ROIs were still identified inside the LAA in many patients (66%), highlighting the fact that conventional ablation approaches do not affect persistent focal or rotational ROIs in the LAA. Moreover, one may speculate that our results suggest focal activation inside the LAA to be one of the dominant mechanisms for the maintenance of AF beyond the PVs or atrial fibrosis. Considering this, further studies are needed to determine if catheter ablation for LAAi in patients with ROIs inside the LAA is safe and effective and may lead to an improvement in terms of freedom from arrhythmia recurrence.

### 4.5. Limitations

This is not an outcome-based study with a limited follow-up duration. It seeks to demonstrate a new method applying bipolar low voltage to quantify the correlation between fibrosis and the presence of focal and rotational activity form CARTOFINDER mapping in patients with PERS. Therefore, the patient numbers and the individual follow-up of the ablation outcome are limited. One very useful outcome of this study is the lack of direct anatomic correlation between the extent of fibrosis from bipolar voltage mapping and ROIs, demonstrating that the amount and distribution of bipolar low voltage could not be used as a surrogate parameter for the presence of ROIs.

## 5. Conclusions

This is the first study reporting data from the novel CARTOFINDER algorithm under routine clinical conditions in an ablation workflow for PERS. ROIs could be discriminated and visualized in all patients. We observed an ablation effect on the presence of ROIs, whereas persistent focal activity was found in certain anatomical segments (i.e., LAA, RA), even beyond PVI and substrate modification. These ROIs might potentially be an additional and individual ablation target when we are able to understand the underlying arrhythmia substrate.

## Figures and Tables

**Table 1:** Patient characteristics (LAD, left atrial diameter; AF, atrial fibrillation; LVEF, left ventricular ejection fraction; DOAC, direct oral anticoagulation). * Left atrial fibrosis was measured using the area measurement tool based on bipolar low-voltage.

Parameter	Patients; *n* = 50
Age (y)	67 ± 11
Male sex (%)	25 (50%)
BMI (kg/m^2^)	29.5 ± 5.4
LAD (mm)	46 ± 7
AF-Duration (months)	30 ± 25
CHA_2_DS_2_-VASc-Score	2.9 ± 1.6
Hypertension (%)	37 (74%)
Diabetes (%)	7 (14%)
Prior stroke (%)	10 (20%)
Heart failure (%)	20 (40%)
LVEF (%)	52 ± 5
Drug therapy	
Antiarrhythmic drugs	10 (20%)
Beta-blocker	39 (78%)
ACE-Inhibitor	39 (78%)
Statin	22 (44%)
Anticoagulation	50 (100%)
Phenprocoumon	5 (10%)
DOAC	45 (90%)
Amount of fibrosis *	
0–10%	2 (4%)
10–20%	2 (4%)
20–30%	17 (34%)
> 30%	29 (58%)

**Table 2:** Procedural data (RA, right atrium; LA, left atrium).

Parameter	Patients; *n* = 50
Fluoroscopy time (min:s)	08:34 ± 04:32
Radiation dose (yGym^2^)	905 ± 763
Procedure time (min)	134 ± 29
Anatomical reconstruction; RA (min:s)	04:23 ± 01:31
Anatomical reconstruction; LA (min:s)	06:29 ± 01:42
CARTOFINDER mapping; RA (min:s)	08:28 ± 04:07
CARTOFINDER mapping; LA (min:s)	13:11 ± 04:16
Ablation time (min:s)	26:35 ± 07:12
Repeat CARTOFINDER mapping; RA (min:s)	05:57 ± 01:08
Repeat CARTOFINDER mapping; LA (min:s)	07:00 ± 02:29
High-density mapping bipolar voltage; RA (min:s)	05:13 ± 02:17
High-density mapping bipolar voltage; LA (min:s)	09:22 ± 04:29
ROIs from CARTOFINDER pre-ablation; LA (*n*)	45 ± 33
ROIs from CARTOFINDER pre-ablation; RA (*n*)	32 ± 19
Mapping points for bipolar voltage; LA (*n*)	2156 ± 1169
Mapping points for bipolar voltage; RA (*n*)	1564 ± 879

## Data Availability

The data presented in this study are available on request from the corresponding author. The data are not publicly available due to privacy and ethical restrictions.
